# Sir B. C. Brodie

**Published:** 1865

**Authors:** 


### Selections.

SIR B. C. BRODIE.—The autobiography of Sir Benjamin C. Brodie, the eminent English surgeon, has just been pupblished in London, and contains, as we might expect in the story of the life of so distinguished a man and scholar, many interesting facts and reflections.

Sir Benjamin was a fourth child, born in 1783. His parents were both superior persons. His father was a clergyman (disappointed it is said, in life, owing to the death of his patron, Lord Holland); his mother, the daughter of a Salisbury banker. Though the family can not be said to have been in straitened circumstances, Mr. Brodie could not afford to send his sons to a public school, and accordingly educated them himself. The family lived a secluded life, principally dependent on themselves for relaxation in the intervals of study; but its members appear to have been united, and the sons were diligent and energetic.

In due time it became proper to shape his education with a view to his future profession. In speaking of this portion of his life, Sir Benjamin expresses some views with regard to natural adaptedness for a calling, and the means by which success is achieved in it, which are somewhat different from the notions that ordinarily prevail:

"As to myself, it was determined that I should embark in some part of the medical profession. Dr. Denman had married one of my father's sisters. Dr. Baillie and Sir Richard Croft had married my first cousins. The great reputation which they had respectively acquired perhaps led my father to give my mind this direction, and disposed me to be easily guided according to his wishes. However that may have been, in the autumn of 1801 I was sent to London, and there entered on those pursuits which have been the chief object of my life. Others have often said to me that they supposed I must have had, from the first, a particular taste or liking for my profession. But it was no such thing; nor does my experience lead me to have any faith in those special callings to certain ways of life which some young men are supposed to have. For the most part these are mere fancies, which are liable to give way to other fancies with as little reason as they themselves first began to exist. Such persons take the *ignotum pro magnifico*, and when they find that the *magnificum* is not equal to their expectations, they as readily fly to something else. The persons who succeed best in professions are those who, having (perhaps from some accidental circumstance) been led to embark in them, persevere in their course as a matter of duty, or because they have nothing better to do. They often feel their new pursuit to be unattractive enough in the beginning, but as they go on and acquire knowledge, and find that they obtain some degree of credit, the case is altered; and from that time they become every day more interested in what they are about. There is no profession to which these remarks are more applicable than they are to the medical. The early studies are, in some respects, disagreeable to all, and to many repulsive. But in the practical exercise of its duties in the hospital there is much that is of the highest interest; and the collateral sciences, to those whose position gives them the opportunity of cultivating them, offer at least as much to gratify our curiosity and excite our admiration as any other branches of knowledge, not even excepting the sublime investigations of astronomy."

In describing an episode of his medical studies, during which he served an apprenticeship in a chemist's shop, he gives us a glimpse of one of the secrets of the profession:—

"During my second as well as my first winter in London, my professional studies were wholly limited to anatomy, except that in the early part of it, and afterwards, when I had no subject for dissection, by Dr. Baillie's advice I attended in a chemist's shop, in order that I might gain some knowledge of the Materia Medica and the making up of prescriptions. The shop was at the corner of Little Newport street, and the proprietor of it was Mr. Clifton, who also practised as an apothecary, exercising his art among tradesmen of the neighborhood. He was an apothecary of the old school, having no science in the ordinary sense of the word; yet, I have no doubt, a useful and successful practitioner. I come to this conclusion because, although there was nothing prepossessing in either his manner or appearance, his practice gradually increased, until at last he was able to give up his shop and live in a large house near Leicester Square, where he dispensed medicines only to his own patients. It is usual in these days to regard this class of practitioners with little respect; but the fact is that they were very useful persons, and, having no very ambitious aspirations, they were within the reach of the poorer orders of society, which is not much the case with the better educated surgeon apothecaries, or, as they are called, general practitioners of the present day, who have expended a considerable sum of money in order to obtain a license to practice. Mr. Clifton's treatment of disease seemed to be very simple. He had in his shop five large bottles, which were labelled *Mistura Salina*, *Mistura Cathartica*, *Mistura Astringens*, *Mistura Chinchonæ*, and another, of which I forget the name, but it was some kind of white emulsion for coughs; and it seemed to me that one of these five bottles he prescribed for two-thirds of his patients. I do not, however, set this down to his discredit; for I have observed that while young members of the medical profession generally deal in a great variety of remedies, they generally discard the greater number of them as they grow older, until at last their treatment of diseases becomes almost as simple as that of the Æsculapius of Little Newport street. There are some, indeed, who form an exception to this general rule, who, even to the last, seem to think that they have, or ought to have, a specific for everything, and are always making experiments with new remedies. The consequence is that they do not cure the patients, which the patients at last find out, and then they have no patients left."

Brodie never seems to have neglected such an important accessory to a professional man's career as general cultivation, which includes mixing in society with the best and most intellectual persons. He marks, as a habit and an advantage, that he preferred the society of persons older than himself, to whom he could look up; in this, how different from the empty persons whose inferiority is gratified by their being "kings of their company!" Nor less instructive is the fact that his manual dexterity was cultivated by great patience and art, instead of being, as many would suppose, a gift of nature. Nothing can be pleasanter than the story of Brodie's steady and increasing successes. It is graced, too, by what may be called vignette characters of his most distinguished medical contemporaries, clever, concise, and liberal in spirit.

